# Eutrophication overrides warming as a stressor for a temperate African seagrass (*Zostera capensis*)

**DOI:** 10.1371/journal.pone.0215129

**Published:** 2019-04-11

**Authors:** Esther F. Mvungi, Deena Pillay

**Affiliations:** 1 Department of Botany, University of Dar es Salaam, Dar es Salaam, Tanzania; 2 Department of Biological Sciences, University of Cape Town, Cape Town, South Africa; University of Sydney, AUSTRALIA

## Abstract

Despite knowledge that seagrass meadows are threatened by multiple global change stressors, significant gaps exist in current knowledge. In particular, little is known about the interactive effects of warming and eutrophication on seagrasses globally, or about responses of African seagrasses to global change, despite these ecosystem engineers providing critical goods and services to local livelihoods. Here, we report on laboratory experiment assessing the main and joint effects of warming and nutrient enrichment on Cape eelgrass (*Zostera capensis*) from the West coast of South Africa, in which morphological attributes, photosynthetic efficiency and elemental content were assessed. Results indicate that shoot density, leaf length, aboveground biomass and effective quantum yield were negatively impacted by both warming and nutrient enrichment. Growth rate, leaf density and leaf width decreased with increasing nutrient levels but not temperature. In addition, epiphytic fouling on seagrass leaves were enhanced by both warming and nutrient enrichment but with warming eliciting a greater response. Collectively, our findings indicate a stronger effect of enrichment on *Z*. *capensis* performance relative to warming, suggesting that the upper levels of coastal eutrophication upon which our experiment was based is likely a stronger stressor than warming. Our findings also highlight limited interaction between warming and nutrient enrichment on *Z*. *capensis* performance, suggesting that effects of these stressors are likely to be propagated individually and not interactively. Our findings raise awareness of susceptibility of *Z*. *capensis* to eutrophication and the need to manage nutrient inputs into coastal ecosystems to preserve meadows of this seagrass and the critical ecosystem functions they provide.

## Introduction

Global change stressors pose significant threats to biodiversity and ecological resilience in marine ecosystems across the planet [1–3]. These stressors do not only impact critical biological and ecological processes [4]; they also negatively feed back to local communities, often in the form of impaired quality of goods and services provided [5–7]. Global warming is a particularly concerning aspect of global change and is commonly considered to be driven by increasing levels of atmospheric greenhouse gases [2], brought on principally by human activities [1–3,8]. The rate at which the planet is heating is alarming [2,3,9,10], with forecasts predicting a rise in global temperatures by 2–4°C by 2100 [3]. Eutrophication, the loading of excessive nutrients into coastal ecosystems, is another dimension of global change that has severe repercussions for biodiversity and ecological integrity [8,11,12]. The intensification of eutrophication over the last few decades is considered a function of increased anthropogenic developments in coastal areas across the globe, with agricultural escalation and fertilizer runoff being particularly important drivers [13,14]. Eutrophication may also indirectly be compounded by global warming due to increased flooding associated with higher precipitation and flooding. While eutrophication management has been employed with some success in developed parts of the world [15], this aspect has been lagging in developing regions.

Global warming and eutrophication, whether acting individually or interactively, are potentially major threats to biodiversity and ecological stability in coastal systems, particularly due to alterations or impairment of ecological functioning in structurally complex coastal ecosystems [2,8,16,17]. These systems are typically dominated by at least one habitat-forming species, which have variously been described as ecosystem engineers, foundation- or keystone-species [4,18,19]. By modulating habitat, refuge and resource availability, structurally complex ecosystems generate disproportionately large effects on regional and local biodiversity [8,20], resulting in conservation agencies often targeting these systems for protection within the broader goal of biodiversity conservation. Such systems are often hierarchically structured, with complex interwoven interactions that in turn confer buffering capacity against ecological change [21,22]. Given the threat to biodiversity posed by losses of structurally complex ecosystems [23], understanding how global change stressors such as warming and eutrophication impact these systems is key for prediction and managing ecological change. Related to this is the need to develop a predictive understanding of (i) whether stressors interact or operate individually, (ii) whether stressors interact additively, synergistically or antagonistically and (iii) the relative strength of stressor effects on structural traits (e.g. density and size) of structurally complex systems and physiological performance, given that these aspects govern to a large degree *per capita* ecosystem engineering activity and hence ecological usage and goods and services provided.

Seagrass ecosystems rank amongst the most important and productive of structurally complex habitats in coastal ecosystems globally [24]. This is due to architecturally mediated enhancement of biodiversity, [25]. Seagrasses are also recognised for the critical ecological functions they provide simultaneously [1,26,27]. In monetary term, goods and services generated from seagrass meadows have an estimated net worth of $1.9 trillion per annum [8]. Unfortunately, seagrass ecosystems rank amongst the most sensitive of structurally complex ecosystems to anthropogenically-induced environmental change, with significant deterioration reported across the globe. Recent syntheses have shed light on the unprecedented impacts of human forcing on seagrass ecosystems, with losses of nearly 30% being reported over the last century [1,3,8,12,18,28]. Rates of seagrass loss rank them amongst the most threatened species on the planet, along with tropical rainforests and coral reefs [8,28,29].

Responses of seagrass ecosystems to anthropogenic and global change stressors have intensified over the last few decades, with several studies quantifying the main effects of warming and eutrophication [30,31]. In this regard, most studies have shown that rising temperatures beyond thermal optima can be detrimental to seagrasses, while eutrophication generally leads to seagrass decline through epiphytic fouling. However, studies testing the interactive effects of these stressors on seagrasses have been relatively rare. This is problematic given recognitions that in nature, processes and stressors often interact to influence ecological functioning, thus requiring studies that examine multi-stressor impacts [32,33]. In parallel, research on anthropogenic and climate-related stressors of seagrasses has lagged significantly on the African continent. This has led to a dearth of (i) quantitative data on how these ecosystems are likely to respond to future global change and (ii) appropriate mitigation plans to preserve ecological functioning that ultimately feeds back to human end users at a regional level. In this paper, we experimentally disentangle the strength of main and interactive effects of warming and eutrophication on the physiological performance (e.g. growth rate, photosynthetic efficiency) of an African seagrass species. *Zostera capensis* is a temperate species that is distributed from the west coast of South Africa to Kenya in East Africa [34]. *Z*. *capensis* is listed as “vulnerable” according to the IUCN red list [34], largely due to rapid rate of its decline, with local estimates indicating a near 38% loss in cover over the past five decades in some systems [4] due principally to anthropogenic activities [12,35]. Such losses are consistent with global trends reporting on the sensitivity of seagrasses to anthropogenic and global change stressors [8,12]. In South Africa (and potentially elsewhere along its range), *Z*. *capensis* is exposed to a range of nutrient levels, with eutrophication being a potentially important stressor. In addition, air temperature data from Langebaan Lagoon on the west coast of South Africa, which is one of two systems still supporting large stands of *Z*. *capensis*, indicate a warming trend over the last 30 years. Based on this, our broader goals were to develop (1) general predictions on responses of *Z*. *capensis* to warming and eutrophication (i.e. elevated water column nutrients) and potential ecological ramifications thereof and (2) contribute to growing knowledge on effects of warming and eutrophication on seagrass ecosystems, which is limited. More specifically, we tested the prediction that elevated nutrients and warming would negatively affect *Z*. *capensis* growth rate, morphological traits (leaf density and dimensions) and photosynthetic efficiency. Effects on morphology were tested to make predictions about structural change in response to warming and eutrophication, while photosynthetic efficiency was assessed to determine seagrass responses at a physiological level.

## Materials and methods

### Experimental design and plant collection

An indoor mesocosm experiment was conducted (aquarium facility in the Department of Biological Sciences, University of Cape Town) in order to achieve our goals. The experiment was based on a fully-factorial design, in which temperature and nutrient levels were manipulated for five weeks to determine their individual and/or interactive effects on seagrass performance. Three temperature levels were used in the experiment viz. ambient (18°C), moderate (24°C), and high (30°C), which were selected to represent the mid to upper temperature ranges experienced within South African estuarine systems [36]. Likewise, the nutrient enrichment treatment comprised three levels viz. no enrichment [N0]; moderate enrichment [N1]: ~ 2 x[NO], and high enrichment [N2]: about 3 to 5 x [NO]. Nutrient levels were within range of those reported for estuaries in South Africa, included highly eutrophic systems [37–41]. All treatments were replicated three times, resulting in a sample size of 27 (3 nutrient levels x 3 temperature levels x 3 replicates = 27) for all response variables, with the exception being that sample size was 18 for phosphate analyses (3 nutrient levels x 3 temperature levels x 2 replicates = 18; details provided further on).

Seagrass was collected from Langebaan Lagoon on the West coast of South Africa (33° 11’ 27” S, 18° 07’ 37”E and 33° 03’ 54” S, 17° 58’ 07” E) during spring low tides in July 2017. Permission to collect seagrass samples was granted by South African National Parks, with Permit number CRC 2016-2017/017–2009/V3. Intact seagrass ramets were harvested using a shovel, cleaned of sediments, stored in plastic buckets and immediately transported to the laboratory. In addition, unvegetated sediment cores (diameter = 10cm, depth = 10cm) were collected from the same area, which acted as a substrate for transplanting seagrasses. In the lab intact and healthy seagrass shoots (of similar length and at least three internodes) were randomly chosen and transplanted into small flower pots (diameter ~ 12.3 cm; depth = ~ 9 cm; 20 shoots/pot) containing previously-collected unvegetated sediment from the field. Six pots containing transplanted seagrasses were arbitrarily distributed among 27 clear plastic aquaria containing seawater (56 l, 2128 cm^2^). The plants were allowed to acclimatize for one week prior to initiation of treatments, at an average water temperature of 15.5°C, salinity 35‰, and the light cycle of 12:12 hours generated by cool white fluorescent light banks (Osram L58/640; Osram L58/965 Biolux; 225 ± 36.5 μmol photon m^-1^.s^-1^ (LI-250A, LI-COR)). Data collected from within each aquarium (i.e. from the 6 pots) were averaged to produce one value.

Temperature and nutrient treatments were randomly assigned to aquaria using the “random” function built into MS Excel. Designated temperature levels were achieved by heating each aquarium individually using aquarium heaters (150–300 W, Eheim Jager and ViaAqua with built-in thermostats) and were monitored using digital thermometers. Nutrient levels were maintained using slow release fertilizer (Osmocote Start), with 50 g and 100 g of fertilizer (for N1 and N2, respectively) filled into bags made of mosquito netting that were mounted on the sides of aquarium. Fresh seawater (1/3^rd^ volume) that was preheated to designated temperature levels was added to each mesocosm daily to simulate flushing and to prevent build-up of toxic chemicals. Nutrient concentrations were assessed in each mesocosm prior to the conclusion of the experiment using a photometer (Hanna Instruments). Mean final loading concentrations ranges for dissolved inorganic nitrogen (DIN) were N0: 745–872 μM; N1: 978–1147 μM; and N2: 1267–1494 μM while phosphate (PO_4_) loading ranges were N0:13–18 μM; N1: 43–55 μM; and N2: 58–79 μM. DIN was calculated based on concentrations of ammonia, nitrite and nitrate that were measured. Algal fouling on the aquaria walls was gently cleaned twice per week.

Physico-chemical properties in aquaria were monitored weekly to ensure consistency in water quality and that designated levels with treatments were being maintained. Light levels were determined 5 cm above seagrass canopies (i.e. below water) using an underwater light meter (LI-250A, LI-COR), while salinity was measured using a hand refractometer (ATIGO S/Mill, 8904 Japan). Salinity was maintained at 35 by adding freshwater whenever needed. pH and dissolved oxygen levels were determined using a water quality multiprobe (Lovibond, SensoDirect 150). Chlorophyll-a concentration was determined using fluorometric analyses (Turner Designs Trilogy; calibrated using de-ionised water) of random water samples collected from aquaria

### Morphometric variables

Growth rate was determined using the punching technique [42]. In brief, five new shoots per pot were randomly selected and punched using a needle above the leaf sheath. After 14 days, the displacement of the scar from the leaf base was recorded and divided by the number of days since punching. Shoot mortality was expressed as the difference in intact shoot numbers per pot between the start and end of the experiment. Number of intact leaves per intact shoot was recorded at the termination of the experiment. Leaf length was determined for the longest second outer leaves of each intact shoot followed by measurements of width at the leaf mid-point. Seagrass biomass was determined at the end of experiment by harvesting seagrass material from each pot, cleaning of sediments, removing epiphytes (which were dried and weighed), separating into below and above ground materials and then drying at 60°C until constant weight was attained.

### Photosynthetic efficiency

Photosynthetic performance of *Z*. *capensis* was assessed on the third week after initiation of treatments using maximum quantum yield (Fv/Fm) and effective quantum yield (ΔF/Fm’) of PhotoSystem II (PS II) through pulse amplitude modulated fluorometry (PAM 2100, Heinz Walz GmbH). Measurements were made on the second outer leaves (second youngest) in order to minimize variability associated with age differences [43]. Maximum quantum yield was determined on three randomly selected shoots per pot, dark adapted for 10 minutes using dark leaf clips (DLC-8, Heinz Walz GmbH) and calculated using the following equation: Fv/Fm = (Fm–Fo)/Fm, after measuring the fluorescence signal from the dark adapted leaf when all reaction centers have opened, using a low intensity pulsed measuring light source (Fo) and during a pulse of saturating light when all reaction centers have closed (Fm). Effective quantum yield was determined by point measurements on ten randomly chosen intact shoots per pot in the illuminated state by applying saturation pulses.

### Elemental chemical analysis

Pooled dried below- and above-ground plant materials were ground using a ball mill. Carbon (C) and nitrogen (N) contents were determined using a CHN analyzer (Department of Archeology; University of Cape Town), whereas phosphorus contents were determined through acid hydrolysis extraction followed by calorimetric analysis (Central Analysis Facility; University of Stellenbosch). Due to funding constraints, phosphate analysis could only be performed on seagrass material from two of the three replicate aquaria per treatment. Thus, there was a sample size of 18 for phosphate analyses (3 nutrient levels x 3 temperature levels x 2 replicates = 18).

### Data analysis

Data were subjected to normality (Shapiro-Wilk test) and homogeneity of variance checks (Levene’s test) prior to analyses. When necessary, data were log_10_ transformed to meet the assumptions of parametric testing. Two-way Analysis of Variance (ANOVA) was used to assess differences in seagrass morphological variables, photosynthetic efficiency and elemental contents between temperature and nutrient treatments, with Tukey *post-hoc* tests employed to detect within-treatment variation. The proportion of variance (PV) accounted for by treatments was calculated by dividing the treatment sum of squares (SS) by the total SS. All analyses were performed using the data analysis platform R studio (R version 3.3.3 of 2017) and the statistical significance levels for all tests were set at p < 0.05.

## Results

### Morphological characteristics

**Fig 1:**
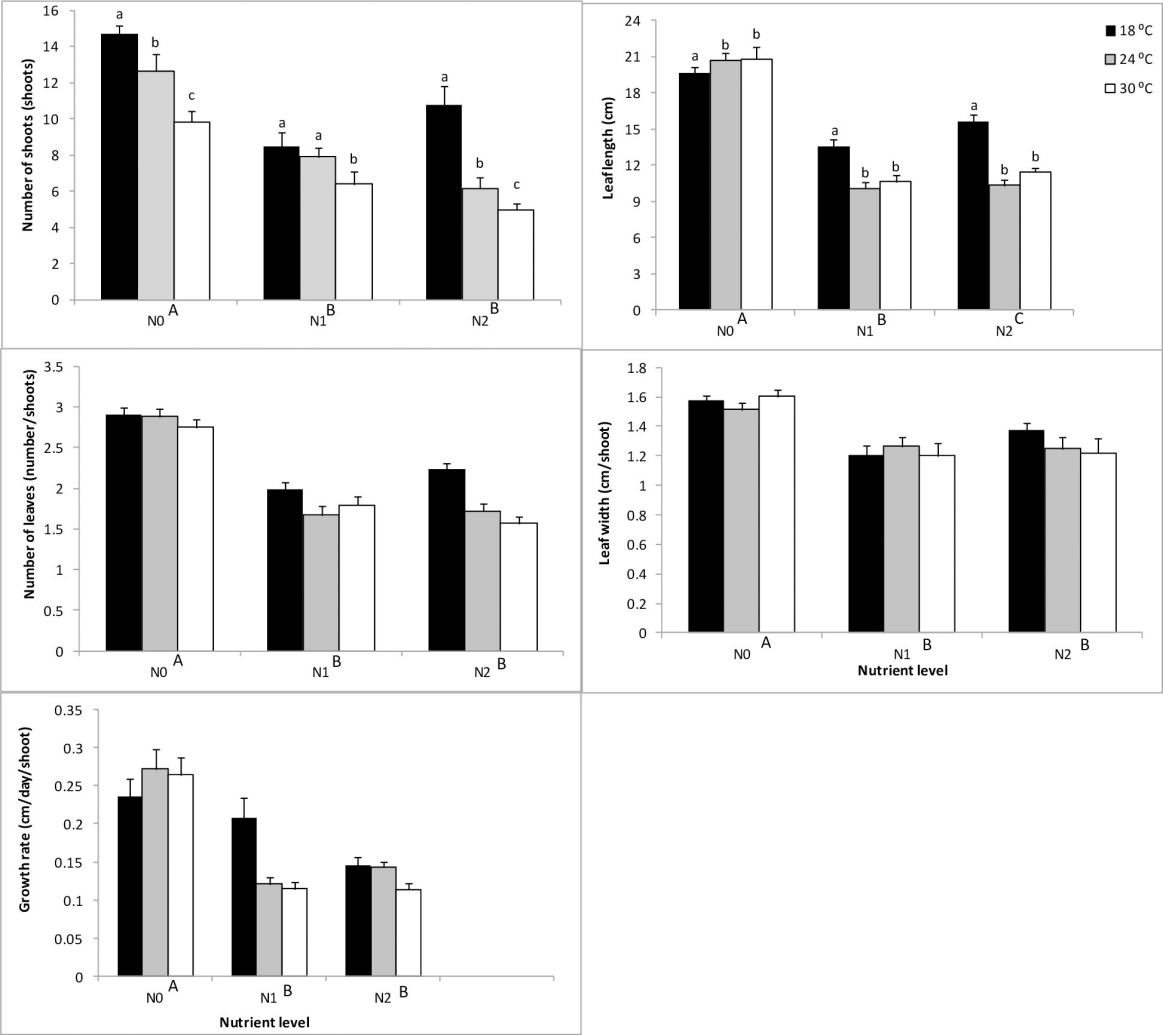
**Main and interactive effects of temperature and nutrient treatments on A: shoot number; B: number of leaves; C: leaf length; D: leaf width and E: growth rate.** Values are mean ± SE, n = 3. Different letters above bars denote differences between temperature levels within nutrient treatments. Different capital letters adjacent to nutrient levels denote differences among nutrient treatments.

Temperature and nutrient levels significantly affected number of shoots at the end of the experiment, which were generally greatest at low nutrient and temperature levels. Up to 75% of shoots died at highest levels of temperature and nutrient relative to the initial number of shoots (Fig 1A; Table 1; S1 Table) relative to low temperature and nutrient treatments. Tukey *post-hoc* testing indicated significant differences (*p* < 0.001) in shoot density between the 18 and 30 ^o^C temperature treatments.

Nutrient treatment was a significant determinant of leaf numbers per shoot, which generally declined at highest levels (Fig 1B). Post hoc tests indicated significant differences (*p* < 0.0001) between enriched (N1 and N2) aquaria and controls (N0).

**Table 1:** Two-way analysis of variance (ANOVA) testing for individual and interactive effects of temperature and nutrient on *Z*. *capensis* performance. AB = Above-ground, BG = Below-ground, PV = Proportion of variance explained by a treatment (only for significant results).

Response Variable	Source of Variation	Df	SS	MS	F- value	P- value	PV
Number of shoots	Temperature	2	82.16	41.08	12.053	0.000	0.27
	Nutrient	2	147.58	73.79	21.650	0.000	0.48
	Temperature:Nutrient	4	17.08	4.27	1.253	0.325	-
	Residuals	18	61.35	3.41			
Number of leaves	Temperature	2	0.447	0.223	2.583	0.103	-
	Nutrient	2	6.570	3.285	8.012	0.000	0.72
	Temperature:Nutrient	4	0.547	0.137	1.583	0.222	-
	Residuals	18	1.550	0.086			
Leaf length	Temperature	2	33.50	16.75	7.90	0.003	0.06
	Nutrient	2	430.90	215.44	101.63	0.000	0.79
	Temperature:Nutrient	4	37.90	9.47	4.47	0.011	0.07
	Residuals	18	38.20	2.12			
Leaf width	Temperature	2	0.010	0.004	0.277	0.762	-
	Nutrient	2	0.609	0.304	16.924	0.000	0.62
	Temperature:Nutrient	4	0.047	0.011	0.664	0.625	-
	Residuals	18	0.324	0.018			
Growth rate	Temperature	2	0.004	0.002	0.747	0.488	-
	Nutrient	2	0.080	0.040	13.132	0.000	0.52
	Temperature:Nutrient	4	0.015	0.003	1.262	0.321	-
	Residuals	18	0.055	0.003			
Root biomass	Temperature	2	0.212	0.106	3.464	0.053	-
	Nutrient	2	0.010	0.005	0.172	0.843	-
	Temperature:Nutrient	4	0.024	0.006	0.201	0.934	-
	Residuals	18	0.552	0.030			
Leaf biomass	Temperature	2	0.038	0.019	4.238	0.031	0.11
	Nutrient	2	0.197	0.098	21.536	0.000	0.57
	Temperature:Nutrient	4	0.030	0.007	1.654	0.204	-
	Residuals	18	0.082	0.004			
Epiphyte biomass	Temperature	2	0.250	0.125	60.129	0.000	0.75
	Nutrient	2	0.031	0.015	7.468	0.004	0.09
	Temperature:Nutrient	4	0.013	0.003	1.561	0.227	-
	Residuals	18	0.037	0.002			
Fv/Fm	Temperature	2	0.001	0.001	1.657	0.219	-
	Nutrient	2	0.000	0.000	0.240	0.789	-
	Temperature:Nutrient	4	0.004	0.001	2.045	0.131	-
	Residuals	18	0.009	0.001			
Yield	Temperature	2	0.042	0.021	6.156	0.009	0.19
	Nutrient	2	0.081	0.041	11.643	0.000	0.36
	Temperature:Nutrient	4	0.042	0.011	3.013	0.045	0.18
	Residuals	18	0.062	0.003			
AG, C contents	Temperature	2	218.00	109.02	5.658	0.012	0.22
	Nutrient	2	295.40	147.70	7.666	0.003	0.24
	Temperature:Nutrient	4	77.90	19.48	1.011	0.427	-
	Residuals	18	346.80	19.27			
BG, C contents	Temperature	2	96.11	48.06	3.853	0.040	0.20
	Nutrient	2	135.26	67.63	5.422	0.014	0.24
	Temperature:Nutrient	4	29.37	7.34	0.589	0.675	-
	Residuals	18	224.53	12.47			
AG, N contents	Temperature	2	0.032	0.015	3.446	0.054	-
	Nutrient	2	0.003	0.001	0.360	0.702	-
	Temperature:Nutrient	4	0.033	0.008	1.820	0.168	-
	Residuals	18	0.083	0.004			
BG, N contents	Temperature	2	0.007	0.003	4.617	0.024	-
	Nutrient	2	0.005	0.002	2.950	0.078	0.23
	Temperature:Nutrient	4	0.005	0.001	1.699	0.194	-
	Residuals	18	0.015	0.000			
AG, C:N ratio	Temperature	2	0.39	0.197	0.066	0.936	-
	Nutrient	2	54.05	27.025	9.090	0.001	0.48
	Temperature:Nutrient	4	5.86	1.464	0.492	0.741	-
	Residuals	18	53.52	2.973			
BG, C:N ratio	Temperature	2	13.95	6.977	0.953	0.404	-
	Nutrient	2	37.58	18.791	2.567	0.104	-
	Temperature:Nutrient	4	18.45	4.611	0.630	0.647	-
	Residuals	18	131.75	7.319			
AG, P contents	Temperature	2	0.000	0.000	0.821	0.470	-
	Nutrient	2	0.004	0.002	39.466	0.000	0.83
	Temperature:Nutrient	4	0.000	0.000	1.515	0.277	-
	Residuals	9	0.000	0.000			
BG, P contents	Temperature	2	0.000	0.000	0.203	0.820	-
	Nutrient	2	0.000	0.000	3.503	0.075	-
	Temperature:Nutrient	4	0.000	0.000	0.708	0.606	-
	Residuals	9	0.000	0.000			
AG, C:P ratio	Temperature	2	33.7	16.8	0.729	0.508	-
	Nutrient	2	1215.3	607.6	26.333	0.000	0.82
	Temperature:Nutrient	4	34.2	8.6	0.371	0.823	-
	Residuals	9	207.7	23.1			
BG, C:P ratio	Temperature	2	45.8	22.9	0.081	0.923	-
	Nutrient	2	2582.3	1291.1	4.544	0.043	0.33
	Temperature:Nutrient	4	2610.6	652.7	2.297	0.138	-
	Residuals	9	2557.3	284.1			

Temperature, nutrient levels and their interaction significantly affected seagrass leaf length, which declined at higher temperatures and nutrient levels (Fig 1). In contrast, nutrient level, but not temperature nor its interaction with nutrients significantly affected leaf width, which generally declined following nutrient addition (Tukey post-hoc p < 0.0001). Similarly, nutrient levels but neither temperature nor their interaction (*p* > 0.05) significantly affected growth rate of the seagrass. Growth rate was greatest in aquarium with no nutrient addition but diminished following nutrient enrichment (Fig 1).

**Fig 2:**
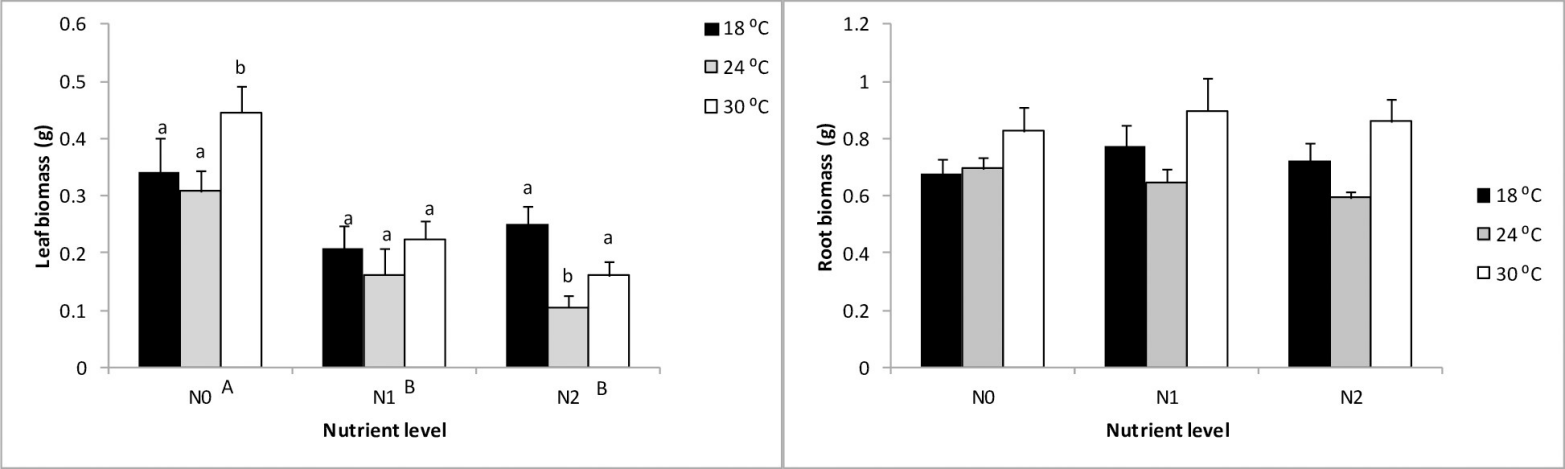
**Main and interactive effects of temperature and nutrient treatments on above ground (A) and belowground biomass (B).** Mean ± SE, n = 3. Different letters above bars denote differences between temperature levels within nutrient treatments. Different capital letters adjacent to nutrient levels denote differences among nutrient treatments.

Seagrass belowground biomass was unaffected by any of the predictors tested (*p* > 0.05), which is probably due to the short duration of the experiment. Temperature and nutrients had significant effects on aboveground biomass, with interactive effects being negligible (Table 1). A decline in aboveground biomass was observed with increasing temperature at high nutrient levels, but this trend was reversed in un-enriched levels, with biomass increasing at the highest temperature. Nutrient enrichment generally resulted in declines in above-ground biomass relative to controls (Fig 2).

**Fig 3:**
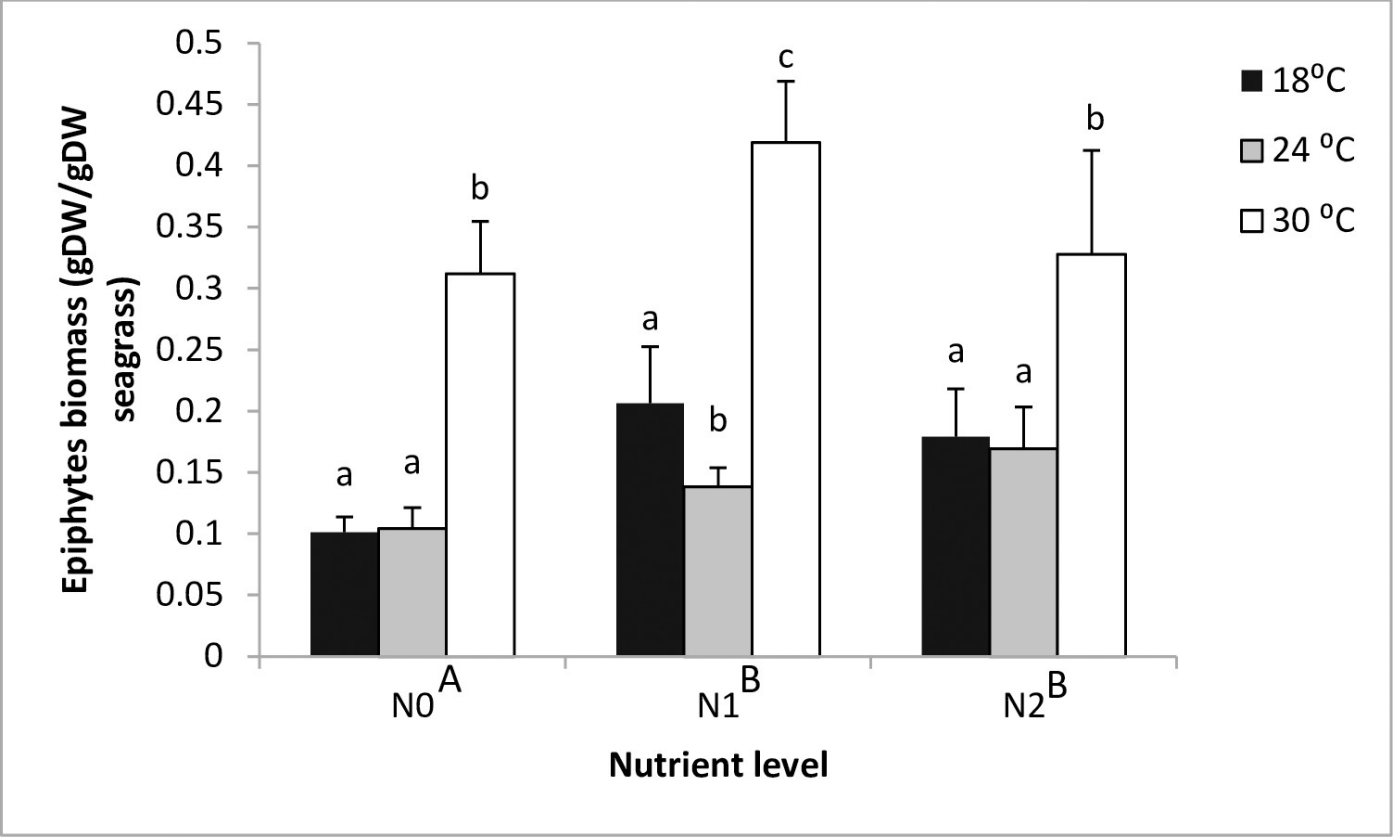
Main and interactive effects of temperature and nutrient treatments on epiphytes biomass. Value are mean ± SE, n = 3. Different letters above bars denote differences between temperature levels within nutrient treatments. Different capital letters adjacent to nutrient levels denote differences among nutrient treatments.

Temperature and nutrients but not their interaction had significant positive effects on epiphyte loads on seagrass blades (Fig 3). Of the two stressors tested, temperature (PV = 0.75) had a stronger effect than nutrients (PV = 0.09) on epiphytic biomass.

#### Photosynthetic efficiency

**Fig 4:**
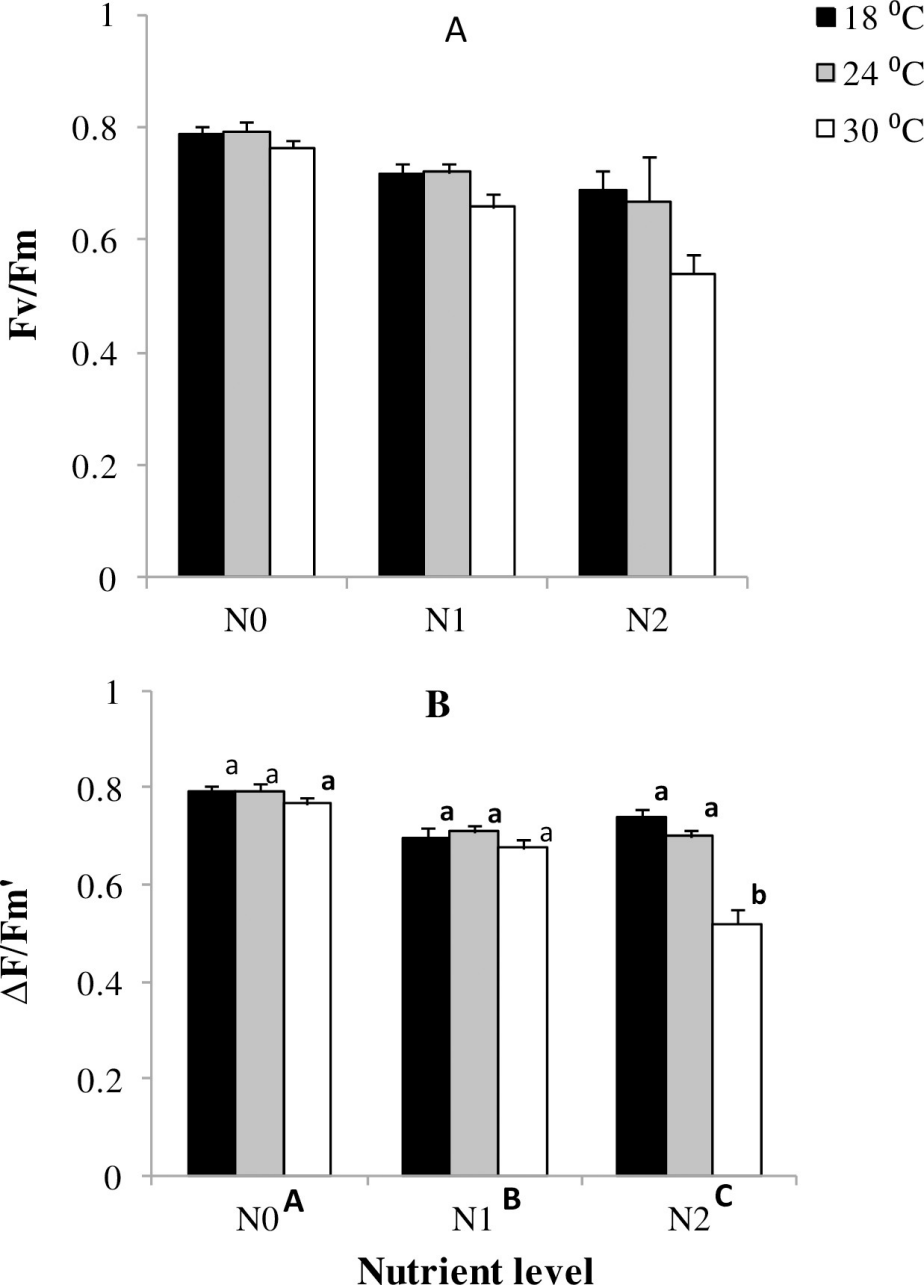
Main and interactive effects of temperature and nutrient treatments on maximum quantum yield and effective quantum yield of PS II in *Z*. *capensis*. Values are mean ± SE, n = 3. Different letters above bars denote differences between temperature levels within nutrient treatments. Different capital letters adjacent to nutrient levels denote differences among nutrient treatments.

Maximum quantum yield in *Z*. *capensis* was not significantly affected by temperature nutrients or their interaction *p* = 0.11, Fig 4). Temperature nutrients and their interaction (Fig 4, Table 1) had significant effects on effective yield, which generally declined with warming and enrichment, with temperature effects being magnified at highest nutrients.

#### Elemental content in seagrass

Aboveground (AG) and belowground (BG) carbon contents were negatively affected by temperature (AG Carbon; BG Carbon and nutrients (AG Carbon *p* = 0.003; BG Carbon but interactive effects were insignificant (*p* < 0.05) (Table 2). AG nitrogen content was not affected by nutrients, temperature or their interaction (*p* > 0.05). BG nitrogen was affected significantly by temperature but not nutrients or its interaction with temperature (*p* > 0.05).

**Table 2:** Carbon, nitrogen, phosphorus contents (% DW) in aboveground (AB) and belowground (BG) parts of *Z*. *capensis* subjected to combined effects of increased nutrient and temperature. T0 = 18°C, T1 = 24°C and T2 = 30°C; N0 = no nutrients, N1 = moderate enrichment, N2 = high enrichment. Mean ± SE.

Treatment Combination	Carbon content (% DW)		Nitrogen content (% DW)		Phosphorus content (% DW)	
	AG	BG	AG	BG	AG	BG
T0N0	35.8 ± 0.90	23.9 ± 0.60	2.82 ± 0.09	1.05 ± 0.03	0.29 ± 0.00	0.16 ± 0.01
T0N1	31.9 ± 0.62	18.2 ± 0.24	3.63 ± 0.31	0.94 ± 0.01	0.38 ± 0.01	0.20 ± 0.00
T0N2	34.6 ± 0.35	20.2 ± 0.61	3.33 ± 0.11	1.04 ± 0.01	0.39 ± 0.01	0.21 ± 0.01
T1N0	37.5 ± 0.30	24.3 ± 0.56	2.91 ± 0.06	1.02 ± 0.08	0.30 ± 0.00	0.18 ± 0.00
T1N1	30.2 ± 1.24	20.4 ± 0.77	3.20 ± 0.22	1.01 ± 0.06	0.36 ± 0.01	0.19 ± 0.00
T1N2	29.9 ± 0.58	19.4 ± 1.84	2.75 ± 0.09	0.90 ± 0.08	0.43 ± 0.03	0.23 ± 0.01
T2N0	34.3 ± 0.83	19.8 ± 0.92	2.59 ± 0.01	0.98 ± 0.05	0.29 ± 0.01	0.19 ± 0.02
T2N1	21.7 ± 3.13	13.1 ± 2.19	2.05 ± 0.26	0.68 ± 0.08	0.41 ± 0.02	0.18 ± 0.03
T2N2	26.4 ± 2.43	18.3 ± 1.30	3.06 ± 0.36	0.87 ± 0.02	0.42 ± 0.01	0.21 ± 0.02

**Fig 5:**
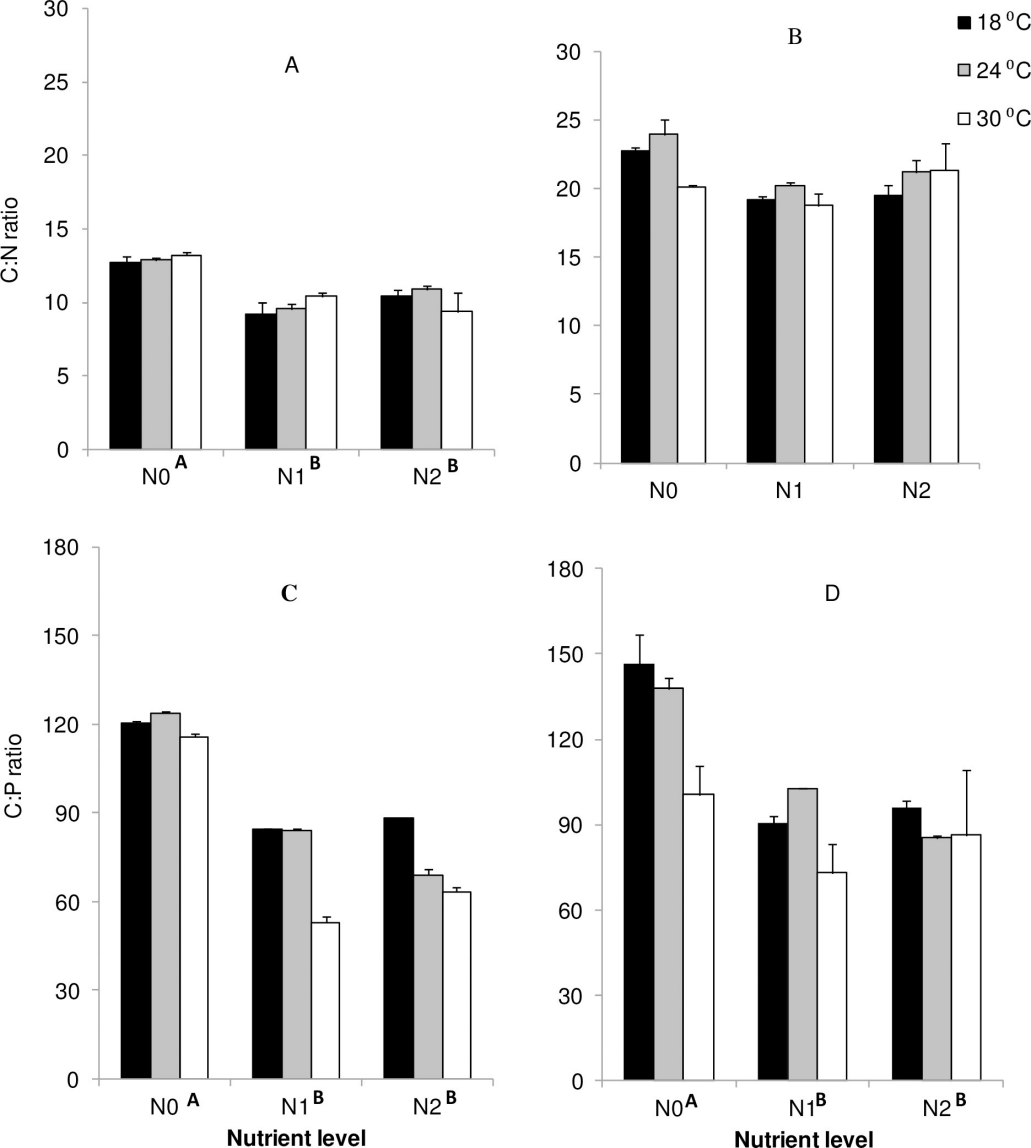
**Main and interactive effects of temperature and nutrient treatments on Carbon: Nitrogen (A & B) and Carbon: Phosphorus (C & D) ratio for *Z*. *capensis*. A & C = aboveground values, C & D = belowground values.** Values mean ± SE. Different capital letters adjacent to nutrient levels denote differences among nutrient treatments.

Phosphorus contents generally increased with nutrient enrichment and temperature, but significant effects were only observed in nutrient enriched treatments for aboveground seagrass sections. AG C:N and C:P ratios decreased with increased nutrient and Fig 5) whereas BG C:N ratio did not vary significantly with either nutrient or temperature (*p* > 0.05).

## Discussion

Knowledge on the individual and interactive effects of elevated temperature and nutrients on seagrass performance is rare (but see [30,31,44,45] for exceptions), despite recognitions that understanding global change impacts requires information on multiple stressor responses and whether such responses are additive, synergistic or antagonistic [2,18,46]. In addition, experimental studies on responses of *Zostera capensis* to abiotic stressors are also limited. In this context, our study has contributed to growing understanding of joint global change stressors on a broadly distributed African seagrass species (*Z*. *capensis*), by quantifying impacts of elevated temperature and nutrient enrichment on its physiological performance. Our results demonstrate significant effects of temperature and nutrient addition on *Z*. *capensis* growth and morphology, but that increasing nutrient levels is a more significant stressor than temperature increases. Evidence for this emanates from 14 of the 20 seagrass response variables being significantly affected by nutrient enrichment compared to 8 variables being affected by warming. Further support arises from estimates of variance explained by treatments (Table 1), which demonstrated stronger effects of nutrient addition for 13 response variables, but only two cases showed greater effects for temperature. In addition, interactions between nutrient enrichment and warming were significant for only two response variables, but even in these cases, nutrient enrichment effects were stronger. Our findings therefore suggest that of the stressors tested and their ranges used in the experiment, nutrient enrichment elicits stronger effects on *Z*. *capensis* performance and that temperature plays a secondary role. Our results also suggest that interactions are likely to be ancillary to main stressor effects, with nutrients and temperature not acting additively or synergistically to impact *Z*. *capensis* physiology. Our findings, however, must be contextualised against the high nutrient levels used in our experiment, which are potentially at the upper end of nutrient values measured in estuaries locally. In the Western Cape of South Africa, our monitoring data indicate nutrient levels as high as 12.62mg/L (PO_4_) and 16.31mg (NH_4_), which are in line with mean values used in the experiment. PO_4_ levels for example ranged between 1.1 and 7.7mg/L across nutrient treatments, while values for NH_4_ ranged between 9.6 and 12.9mg/L.

Nutrient enrichment (primarily) and warming, especially at the higher levels, generated detrimental effects on seagrass morphology (growth rate, above-ground biomass and leaf and shoot density and size), photosynthetic performance and elemental concentrations. Such responses are likely a product of inherent biological traits of *Z*. *capensis* (e.g. thermal tolerance), which ultimately determine physiological response thresholds to environmental conditions and stressors [47]. A low thermal tolerance for example, would imply a low response threshold to increasing temperatures. Such thresholds are likely to be fluid to a degree and dependent on environmental contexts. Our findings would suggest that the mid- to upper ranges of stressors used in this experiment exceed the nutrient and temperature optima for *Z*. *capensis*, thus leading to impaired physiological performance [48–50]. Our findings also concur with similar studies on temperate seagrasses that have demonstrated detrimental effects of increasing nutrients and temperature on physiological performance [44,45]. In terms of temperature tolerance specifically, studies have shown that levels beyond roughly 25 ^o^C generates adverse effects on temperate seagrasses while tropical species could survive up to 40 ^o^C [14,49–52]. In the present study, being a temperate species, *Z*. *capensis* showed significant deterioration at intermediate (24 ^o^C) and high (30 ^o^C) temperature levels. It must be emphasized however, that thermal tolerance could be higher in *Z*. *capensis* stands towards the northern limits of its distribution.

Complex physiological mechanisms and feedbacks are likely to underpin net stressor effects observed on *Z*. *capensis* in our experiment. While we lack the data to explicitly invoke these mechanisms as drivers of seagrass response, such mechanisms have been studied in the literature on stressor effects on seagrass physiology. Firstly, *Z*. *capensis* may incur energetic costs in taking up excessive nutrients such as nitrates, potentially due to limited or no uptake feedback mechanisms, as reported for other seagrass species [53,54]. In addition, elevated temperature and high nutrient levels in sediments may lead to the development of anoxia/hypoxia and elevated concentrations of toxic hydrogen sulphide in the rhizosphere [55]. Likewise, increasing temperature beyond thermal optima can also impair nutrient uptake processes [53,56]. The reductions in growth properties (growth rate, above ground biomass, leaf dimensions and density) of *Z*. *capensis* that we recorded could therefore be a product of nutrient enrichment (primarily) and warming (secondarily) effects impairing physiological functions [49,52]. Warming may also limit photosynthetic yield by impacting enzyme functioning required for photosynthesis and similarly reduce growth properties [50,57,58]. It is theoretically conceivable that lowered photosynthetic yield and reductions in *Z*. *capensis* growth create mutually re-enforcing negative feedback loops that accelerate reductions of yield and growth [50,58,59]. This is plausible given that declining growth is generally associated with reduced surface area available for photosynthesis, which in turn further reduces growth. Overall, trends in C:N ratios indicate a decline in tissue carbon content in response to increasing warming and nutrients. Lastly, one of the most important mechanisms by which nutrient enrichment and warming initiate deterioration in *Z*. *capensis* physiological performance is by increasing fouling by epiphytic algae, which was strongly demonstrated in our experiment. It is generally accepted that fouling reduces light available for photosynthesis leading to declining physiological tolerance and localized extinction in extreme cases [14]. Data from field studies indicate that an excess of nutrients in coastal ecosystems drives fouling and hence declines in seagrasses [1]. Data from experimental studies are limited, with studies showing positive, negative and neutral effects of nutrients on fouling levels [60,61]. An interesting finding emanating from our experiment was that although nutrients had a positive effect on epiphytic loads, as predicted by several contemporary field studies, the effect size was outweighed by warming. Our finding that warming can elevate seagrass fouling is supported by [44] and is consistent with observations that elevated temperatures can favor proliferation of micro- and macrophytes colonizing leaf blades [60]. Our findings suggest that warming may potentially be a stronger driver of epiphytic fouling on *Z*. *capensis* than eutrophication, but that nutrient enrichment is a stronger general stressor for other physiological processes. However, caution should be exercised when interpreting findings related to fouling in our experiment, given that it was conducted in a grazer free context. In the presence of grazers, warming could for example indirectly reduce fouling levels by increasing effectiveness of ectothermic grazers. In addition, the stronger effect of temperature on fouling may be due to the very high nutrient levels used in the study, due to there being no nutrient limitation.

Our findings of nutrients (primarily) and warming inducing declines in *Z*. *capensis* growth is consistent with the notion of seagrasses being displaying morphological plasticity in response to environmental conditions. [61,48]. *In situ* studies have for example reported reductions in leaf sizes of *Zostera japonica* and *Z*. *noltii* from subtidal to high intertidal habitats [62–64] presumably in response to changing abiotic stress. The changes in growth of *Z*. *capensis* in response to experimental warming and nutrient enrichment may thus indicate some degree of plasticity in response to environmental variability.

### Ecological implications of findings

While our data highlight the important individual effects nutrient enrichment and increasing temperatures can have on *Z*. *capensis*, they also have key implications for predicting changes in regional ecosystem functioning in the face of global change. Our data indicate that increasing levels of nutrients are primarily likely to lead to shifts in architectural attributes of *Z*. *capensis*, principally involving reductions in overall plant size, density and growth rate, which are likely in turn to induce cascading alterations to multiple ecological functions and processes [8,16,65,66]. In the long-term, warming-induced fouling may induce similar shifts by negatively affecting photosynthesis. Structural traits of seagrasses have, for example, been shown to influence spatial habitat use by inhabitants by altering (i) susceptibility of residents to predation, (ii) the quality of refuge provision against abiotic stress and (iii) availability of trophic resources [21,22]. Reductions in coastal *Z*. *capensis* populations through nutrient enrichment and warming (secondarily) may also potentially reduce spatial subsidies to adjacent ecosystems, and the potentially positive bottom-up effects on consumers [2,8,16,65,66]. All of these concerns speak to the need for mitigation measures to protect *Z*. *capensis* from eutrophication (primarily) and warming induced stress along. Central to mitigation would be the need to reduce nutrient inputs into coastal ecosystems. Education programs and greater awareness have been linked to reductions in nutrient inputs into coastal ecosystems, for example [15]. However, mitigation in developing countries is problematic, due to burgeoning population growth, development and poor infrastructure leading to major eutrophication problems. Restoration/rehabilitation of fringing wetlands and marshes is also necessary for excessive nutrients to be absorbed by producers. Lastly, restoration of epiphytic grazing species like gastropods, is essential in protecting *Z*. *capensis* from the harmful effects of fouling induced by both warming and eutrophication [15]. In South Africa for example, grazers such as the seagrass limpet (*Siphonaria compressa*), have become critically endangered. Their protection will not only positively affect their conservation status, but also benefit seagrasses in the long-term. Such mitigation actions may thus lead to mutually reinforcing positive feedbacks that benefit both seagrasses and *S*. *compressa*.

## Supporting information

S1 Table Supplementary file with data set used for analysis. (XLSX)
